# Thrombosis in Cerebral Aneurysms and the Computational Modeling Thereof: A Review

**DOI:** 10.3389/fphys.2018.00306

**Published:** 2018-04-04

**Authors:** Malebogo N. Ngoepe, Alejandro F. Frangi, James V. Byrne, Yiannis Ventikos

**Affiliations:** ^1^Department of Mechanical Engineering, University of Cape Town, Cape Town, South Africa; ^2^Centre for High Performance Computing, Council for Scientific and Industrial Research, Cape Town, South Africa; ^3^Stellenbosch Institute for Advanced Study, Wallenberg Research Centre at Stellenbosch University, Stellenbosch, South Africa; ^4^Center for Computational Imaging and Simulation Technologies in Biomedicine, University of Sheffield, Sheffield, United Kingdom; ^5^Department of Neuroradiology, John Radcliffe Hospital, Oxford, United Kingdom; ^6^UCL Mechanical Engineering, University College London, London, United Kingdom

**Keywords:** cerebral aneurysm, thrombosis, flow diverter, interventional planning, computational modeling

## Abstract

Thrombosis is a condition closely related to cerebral aneurysms and controlled thrombosis is the main purpose of endovascular embolization treatment. The mechanisms governing thrombus initiation and evolution in cerebral aneurysms have not been fully elucidated and this presents challenges for interventional planning. Significant effort has been directed towards developing computational methods aimed at streamlining the interventional planning process for unruptured cerebral aneurysm treatment. Included in these methods are computational models of thrombus development following endovascular device placement. The main challenge with developing computational models for thrombosis in disease cases is that there exists a wide body of literature that addresses various aspects of the clotting process, but it may not be obvious what information is of direct consequence for what modeling purpose (e.g., for understanding the effect of endovascular therapies). The aim of this review is to present the information so it will be of benefit to the community attempting to model cerebral aneurysm thrombosis for interventional planning purposes, in a simplified yet appropriate manner. The paper begins by explaining current understanding of physiological coagulation and highlights the documented distinctions between the physiological process and cerebral aneurysm thrombosis. Clinical observations of thrombosis following endovascular device placement are then presented. This is followed by a section detailing the demands placed on computational models developed for interventional planning. Finally, existing computational models of thrombosis are presented. This last section begins with description and discussion of physiological computational clotting models, as they are of immense value in understanding how to construct a general computational model of clotting. This is then followed by a review of computational models of clotting in cerebral aneurysms, specifically. Even though some progress has been made towards computational predictions of thrombosis following device placement in cerebral aneurysms, many gaps still remain. Answering the key questions will require the combined efforts of the clinical, experimental and computational communities.

## Introduction

**Figure 1:**
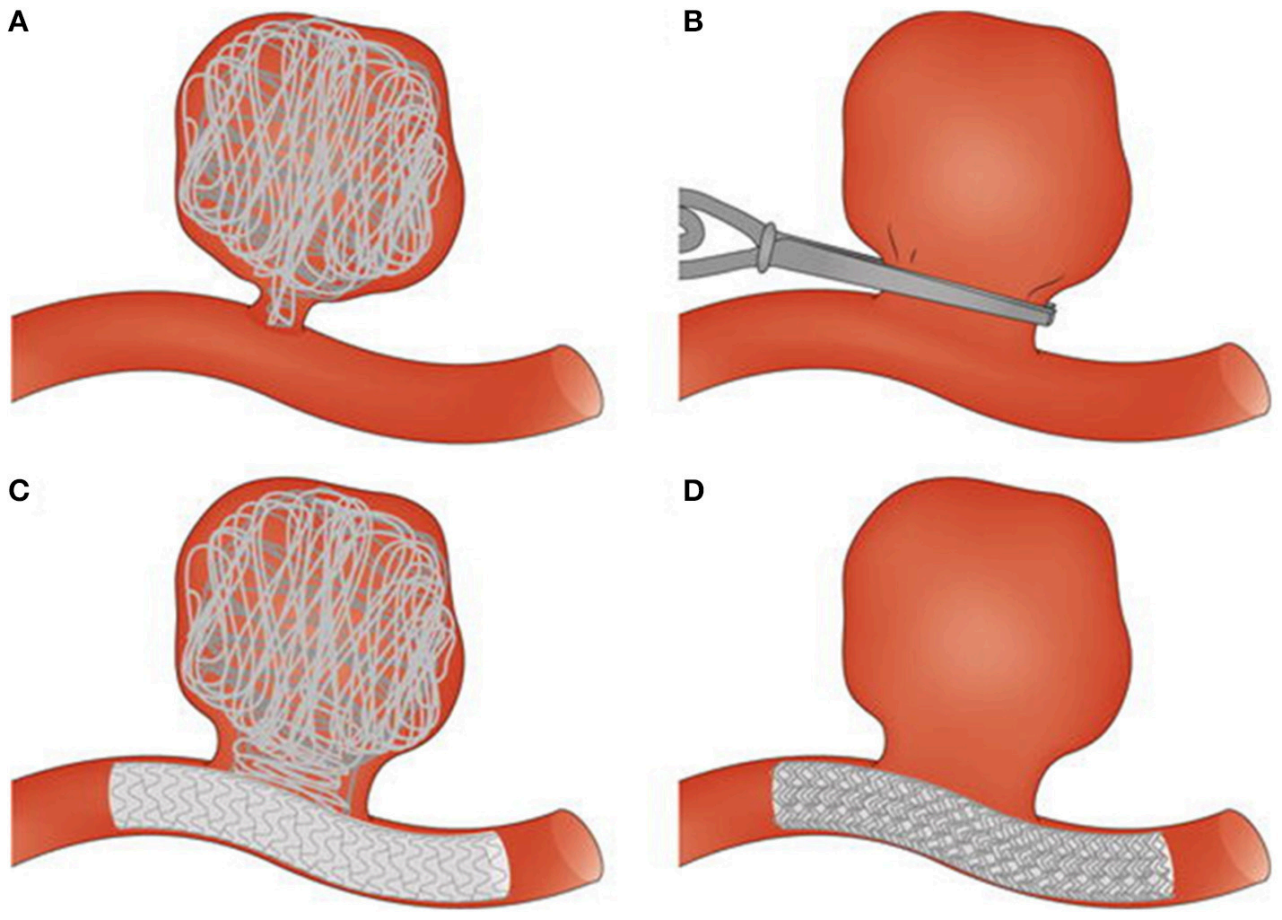
Surgical and endovascular treatments for cerebral aneurysm thrombosis. **(A)** Endovascular coiling of the aneurysm sac. **(B)** Surgical clipping of the aneurysm neck. **(C)** Endovascular treatment combining use of coils and a stent. **(D)** Endovascular treatment with a flow diverter. Taken from Perrone et al. (2015).

The hemostatic process maintains the integrity of the circulatory system. Under physiological conditions, clotting occurs following injury to a blood vessel. This overlap of platelet activity and fibrin formation ensures that bleeding is arrested and initiates the healing process. In some individuals, the balance between the procoagulant and anticoagulant processes is disturbed, resulting in too much clotting or in other cases, insufficient clotting. There are also pathologies, such as cancers and acute coronary syndromes, where clotting occurs with no injury to the blood vessel wall (Ruf and Mueller, 2006; Cimmino et al., 2011). For both pathologies, clot development has been linked to circulating forms of tissue factor found on tumor cells or cell-derived macroparticles. Thrombosis is a biological response closely linked to cerebral aneurysms, which are balloon-like dilations of blood vessel walls caused by weakening of the vessel wall layers (Lawton et al., 2005). In studies covering populations in the United States, Canada, Europe and Japan, it was found that cerebral aneurysms have a prevalence of 1–5% and rupture risk of 0.6% per annum, with 30–50% subsequent mortality or severe morbidity (Wiebers, 2000; Wermer et al., 2007). Thrombosis has been observed in both ruptured and unruptured aneurysms (Eller, 1986; Ishikawa et al., 2006; Calviere et al., 2011). In unruptured cases, thrombosis can either stabilize the aneurysm or accelerate the path to rupture. This is true both for spontaneous clots, which are in the aneurysm sac with no external input or interference, and for device-induced clots (Whittle et al., 1982; Byrne et al., 1997, 2010; Vanninen et al., 2003; Cohen et al., 2007). The different surgical (clips) and endovascular (coils, coils and stent, flow diverter) treatments that result in device-induced clotting are illustrated in Figure 1 (Perrone et al., 2015). Given the important contribution that clots make to aneurysm stability, thrombosis outcome following device placement could give strong clues about eventual unruptured aneurysm outcome. The ability to predict unruptured aneurysm progression is increasingly important, as advances in medical imaging technology have increased the number of aneurysms identified accidentally during routine scans or examinations for other conditions (Rinkel et al., 1998; Winn et al., 2002; Steiner et al., 2013). The decision to treat an aneurysm which would otherwise have remained harmless throughout the life of the patient in question places an unnecessary burden on healthcare systems and introduces iatrogenic risks (Wardlaw and White, 2000). Conversely, the decision not to treat a seemingly asymptomatic aneurysm may later prove fatal should the aneurysm rupture. Decisions regarding intervention are made based on morphological descriptors of the aneurysm. Factors such as size, location, shape, aspect ratio, and bottleneck factor are considered when deciding whether treatment is necessary (Wiebers et al., 2003; Morita et al., 2005; Raghavan et al., 2005; Wiebers, 2005; Wermer et al., 2007; You et al., 2010).

Owing to the patient-specific nature of cerebral aneurysms, virtual interventional planning tools capable of predicting outcome on a personalized basis are highly desirable. Various computational tools predict the effect of interventional device placement (Kakalis et al., 2008; Chong et al., 2014; Peach T. et al., 2014). Often, these tools and models are designed for use in a patient-specific, interventional planning environment. Many models focus on predicting the altered hemodynamic conditions following intervention. A small subset of these have focused on cerebral aneurysm thrombosis, given that in this particular pathology, clotting outcome has such a significant impact on eventual cerebral aneurysm evolution (Ouared et al., 2008; Rayz et al., 2010; Peach T. W. et al., 2014; De Sousa et al., 2015; Ngoepe and Ventikos, 2016; Ou et al., 2016). The ideal computational tool would be capable of predicting both the hemodynamic changes and clot maturation post endovascular device placement, and would determine the best treatment course for an individual. Such a tool would also be useful for optimizing anticoagulant adjunct treatments on a per patient basis.

This review aims to examine the various aspects of thrombus development in unruptured cerebral aneurysms, with particular focus on those that are of relevance to developing virtual interventions. The first part of the review presents and introduces the mechanisms of cerebral aneurysm thrombosis based on current understanding of physiological clotting, i.e. desirable clotting that occurs following injury to a blood vessel. The second part explores insights gained from clinical observations. The third part highlights the demands placed on a computational model of cerebral aneurysm thrombosis if it is to be used in an interventional planning context. The final part presents existing computational models of thrombosis and of thrombosis in cerebral aneurysms. The conclusion then highlights progress made and future direction.

## Mechanisms of cerebral aneurysm thrombus development - building up a framework from biochemistry and platelet biology

From a biochemical and platelet biology viewpoint, physiological clotting is the foundation from which current understanding of unruptured cerebral aneurysm thrombosis is developed. The rationale for making use of these physiological models is explained in the first part of this section, followed by the similarities and differences between the two processes.

At present, the exact process of cerebral aneurysm thrombosis is poorly understood. Experimental studies have focused on the biological processes that unfold after a stable clot has formed (Bouzeghrane et al., 2010). *In vivo* observations of clot formation have proven challenging because of the multiscale nature of the process (Falati et al., 2002). Given these constraints, it is necessary to consider the factors that would contribute to thrombosis in general and to then construct an understanding of cerebral aneurysm thrombosis from that basis. Virchow postulated that clotting depends on blood composition, the state of the vascular endothelium and the local hemodynamics (Virchow, 1856). It is generally accepted that in cerebral aneurysms, the main deviations from the physiological state relate to the state of the vascular endothelium and local hemodynamics (because of geometric changes) (Stehbens, 1963; Scanarini et al., 1978; Humphrey and Canham, 2000; Rajesh et al., 2004). There is little evidence to suggest that blood composition is altered dramatically in this particular pathology, as aneurysms are more a disease of the blood vessel than of the blood. For cerebral aneurysm thrombosis, pathways relating to platelets and coagulation proteins are based on physiological clotting pathways, which have been thoroughly explored by the biochemistry community (Davie and Ratnoff, 1964; Macfarlane, 1964; Furie and Furie, 1992; Falati et al., 2002; Hoffman, 2003). The ensuing discussion in this section presents the putative similarities and key differences between physiological and cerebral aneurysm clotting pathways. These details are important in developing a computational cerebral aneurysm model that moves beyond hemodynamic considerations only.

For both physiological clotting and cerebral aneurysm thrombosis, clot development is an overlap of spatial and temporal processes involving platelets, coagulation proteins and anticoagulant mechanisms. Injury to the vessel wall results in expression of von Willebrand's factor (vWF), which enables platelet attachment to the subendothelial collagen layer (Sakariassen et al., 1979; Stel et al., 1985). Platelets are recruited to the site of injury to prevent further blood extravasation and to facilitate the healing process. Haemodynamics and the presence of blood cells affect the transport of platelets to the site of injury and interaction of those platelets with the vessel wall (Goldsmith and Karino, 1987). Platelet shape changes are observed during thrombogenesis and the key stages include deposition, activation, adhesion and aggregation (Kuwahara, 2002). Upon initial contact with the injury zone, platelets take on a rolling-ball shape and extrude filaments along their surface. Interactions between the platelet's receptor GP Ib and vWF result in a gradual change towards a hemispherical shape. The same interactions result in firm, reversible adhesion arising from the activation of the platelet's integrin α_IIb_β_3_. The interaction between this activated integrin and vWF results in the formation of permanent, irreversible bonds. This comes with extensive spreading of the platelet. Activated platelets can recruit other platelets to the site of injury and this aggregation results in the formation of a platelet plug.

**Figure 2:**
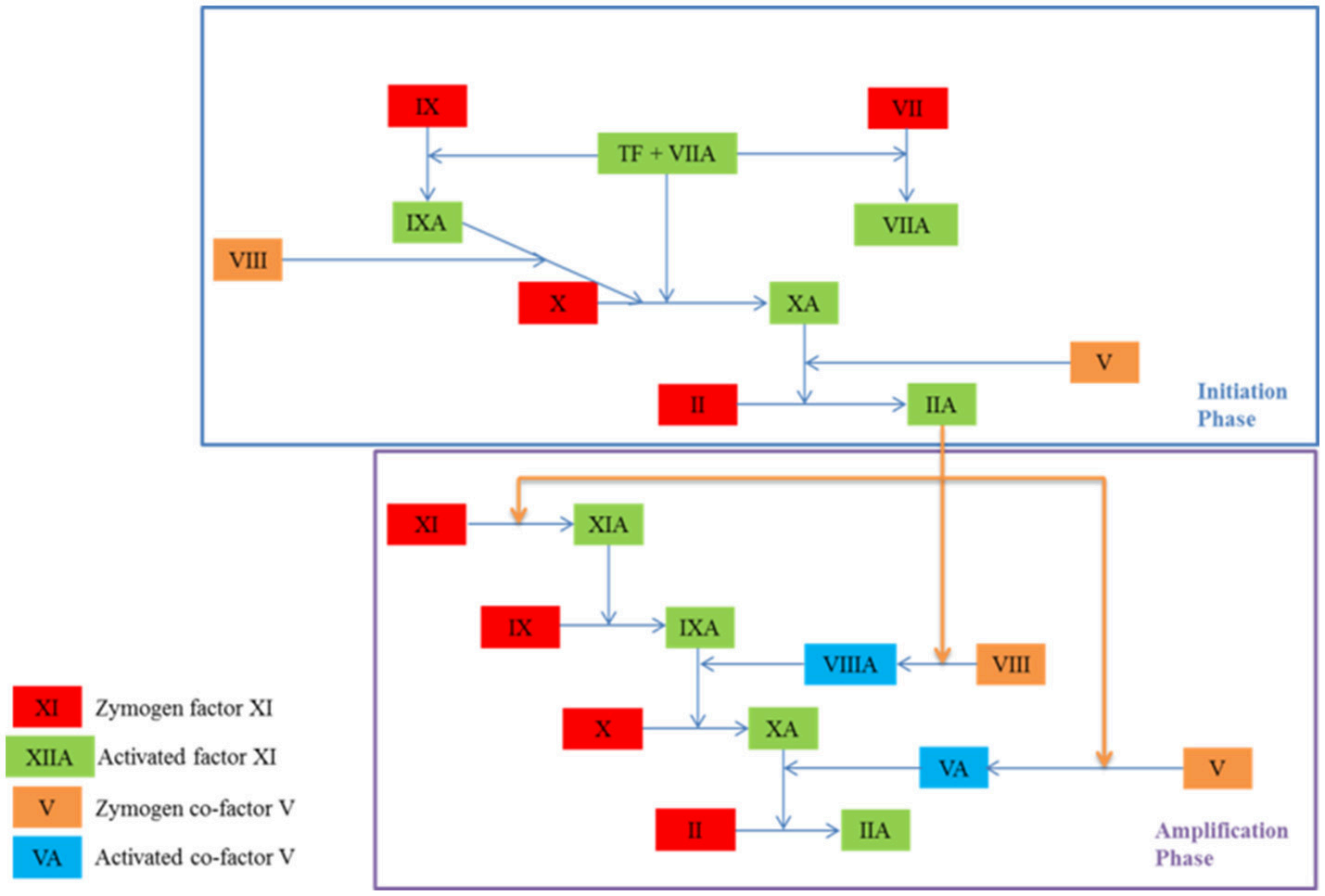
The initiation and amplification phases of coagulation which result in the formation of thrombin (IIA), an enzyme which catalyses the formation of fibrin. A coagulation protein circulates in the blood in an inactive state known as a zymogen and contributes to the clotting process once activated. Co-factors amplify and accelerate the production of activated proteins. They become active in the amplification phase.

A cascade of coagulation reactions occurs alongside platelet activity, resulting in the formation of a fibrin mesh that secures the platelet plug in place until healing has occurred. This process is characterized by clot initiation, and then amplification and propagation, as seen in Figure 2 (Falati et al., 2002; Hoffman, 2004; Furie and Furie, 2005). The exposure of tissue factor initiates the reactions and results in the formation of a small amount of thrombin (Orfeo et al., 2005). During amplification, this small quantity of thrombin activates platelets and co-factors V and VIII (Hoffman, 2004). The activation of these cells and proteins ushers in the propagation phase, which results in the accelerated production of thrombin and platelet recruitment. During propagation, some reactions are limited to platelet membranes, thus regulating and limiting the process to the injury site (Panteleev et al., 2006). Thrombin is a key protein in clot development as it acts as an enzyme for the reaction which results in the formation of fibrin (Mann et al., 2003). It is also responsible for the activation of platelets and is easily measurable. Tests of clotting function determine the efficacy of an individual's clotting mechanism by measuring thrombin. Many of the coagulation reactions take place on platelet membranes or on subendothelial cell membranes (Brinkman et al., 1994). Platelets therefore play a key role not only in filling the gap created by the injury, but also in supporting some of the enzymatic reactions which result in the formation of a fibrin mesh (Rosing et al., 1985).

**Figure 3:**
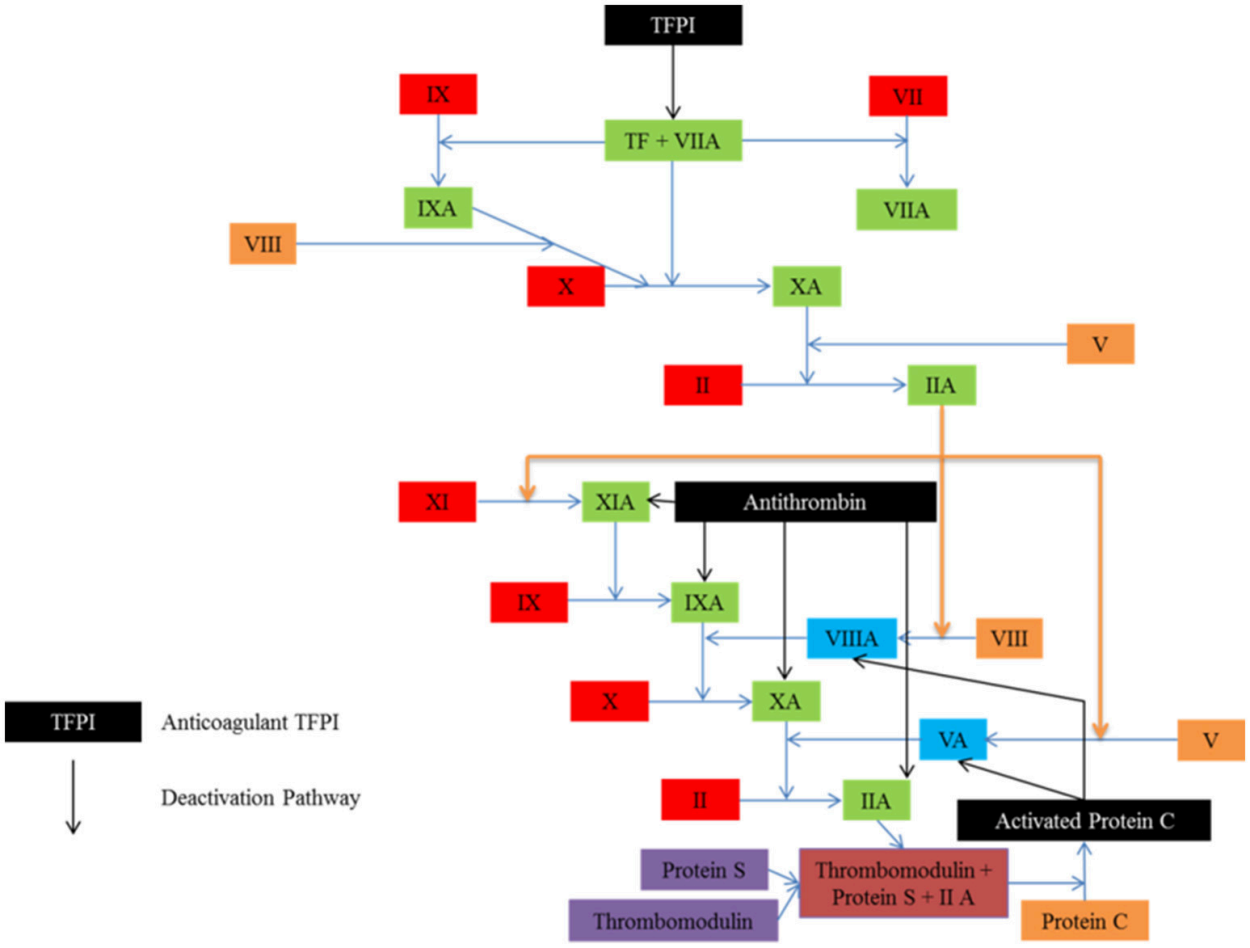
Several anticoagulant mechanisms exist to ensure that clotting is limited to where it is needed. Tissue factor pathway inhibitor (TEFI) inhibits tissue factor, the protein responsible for clot initiation. Antithrombin targets the activated factors, while activated protein C inhibits the activated co-factors.

To ensure that clotting is limited to where it is needed only, both in time and space, several anticoagulant mechanisms limit the process. These can be seen in Figure 3. The endothelial layer expresses substances such as nitric oxide, preventing spontaneous clotting where there is no injury (Bunting et al., 1976; Ignarro et al., 1987; Cines et al., 1998). In addition, once clotting has taken place, antithrombin deactivates key proteins such as thrombin, and tissue factor pathway inhibitor destroys the efficacy of tissue factor (Seegers et al., 1953; Abildgaard, 1968; Yin et al., 1971; Damus et al., 1973; Kurachi et al., 1976; Di Scipio et al., 1978; Rao and Rapaport, 1987).

The key differences between physiological clotting and thrombosis in cerebral aneurysms relate to initiation. For both processes, tissue factor must initiate the series of clotting reactions that result in the formation of a fibrin mesh, however the source of this tissue factor is thought to differ (Bugge et al., 1996; Carmeliet et al., 1996; Toomey et al., 1996; Hoffman, 2003, 2004; Hoffman et al., 2006; Mann, 2006; Furie and Furie, 2008; Morel et al., 2011). In traditional coagulation models, injury to the vessel wall is necessary for the exposure of subendothelial tissue factor, which initiates clotting. In cerebral aneurysms, specifically, the vessel wall is quite compromised with the endothelium absent for large portions of the sac (Humphrey and Canham, 2000). Clot initiation has been linked to this endothelial damage present in the aneurysm sac (Sutherland et al., 1982). However, fairly recent studies have also verified the presence of blood-borne tissue factor, a circulating form of the protein that contributes to the clotting process (Giesen et al., 1999; Hathcock and Nemerson, 2004; Morel et al., 2006). The exact role of blood-borne tissue factor in clotting is contested. There seems to be some consensus that this circulating form of tissue factor has an influence where there is an abnormal stimulus, such as increased shear rate (Butenas et al., 2005, 2009; Hoffman et al., 2006; Mann, 2006; Morel et al., 2006; Okorie et al., 2008). This is significant for cerebral aneurysms as the condition often results in complex hemodynamics with varying shear rates. It has been shown that under flow conditions, adding circulating tissue factor to blood amplifies the production of fibrin (Okorie et al., 2008). It would therefore be beneficial to determine the exact shear rate threshold at which circulating tissue factor becomes important and to consider this when examining cerebral aneurysm hemodynamics.

The second key difference relates to the altered pathological hemodynamics and their effect on platelet activity. In arterial diseases arising from stenosis, platelets can be activated by abnormal flow patterns and high shear stresses (Einav, 2004). Activated platelets can attract other platelets, resulting in the formation of platelet plugs. The prominence of recirculation zones common in disease encourages mixing of coagulation proteins, thus creating an enabling environment for fibrin formation (Rayz et al., 2008; Di Achille et al., 2014; Peach T. et al., 2014).

Considering all the contributing factors and gaps in existing knowledge, it is reasonable to base our initial understanding of cerebral aneurysm thrombosis on the physiological clotting process. The platelet and biochemical pathways describing the latter can therefore be used as a framework for developing a cerebral aneurysm thrombosis model that accounts for platelet activity and/or coagulation proteins. The main differences relate to initiation and clot maturation (Kadirvel et al., 2014; Gester et al., 2016). The relative contributions of subendothelial and blood-borne tissue factor must be considered carefully. In addition, the hemodynamic environment must be well characterized as disturbances in flow have a significant impact on platelet activation and protein transport.

## Insights from clinical observations of thrombosis in cerebral aneurysms

Our current understanding of some of the general features which are unique to unruptured cerebral aneurysm thrombosis in humans has been developed from clinical observations. In this section, observations made following coiling and flow diversion are presented. Most of the studies included here are those that were evidence for the American Heart Association/American Stroke Association “Guidelines for the Management of Patients With Unruptured Intracranial Aneurysms” (2015) and the European Stroke Organization “Guidelines for the Management of Intracranial Aneurysms and Subarachnoid Hemorrhage” (2013) (Steiner et al., 2013; Thompson et al., 2015). With regards endovascular treatment, the guidelines focused on coil occlusion and referred to a few articles on emerging technologies, of which flow diversion was one. In this section, additional studies were also considered for flow diversion. Given the relative novelty of flow diversion as a viable treatment option, the criteria ensured that the results of the early clinical studies conducted were included at least once, either directly or in a meta-analysis. Criteria to include these additional studies (reviews or original research) were (a) at least nine-hundred unruptured cerebral aneurysms, (b) treatment with flow diverters, (c) published between 2013 and 2015 and (d) reporting of occlusion outcome. Most clinical studies that focus on endovascular treatment infer clotting outcome from occlusion of the aneurysm sac following intervention. Clotting is reported as full or partial occlusion in the two subsections that follow. To conclude this section, the limitations of using occlusion as a measure of clotting outcome are discussed and the key clinical questions that relate to thrombosis in aneurysms following endovascular intervention are highlighted. The desire to answer these questions has driven the approaches used when developing computational models for thrombosis in cerebral aneurysms following device placement.

### Coiling in unruptured aneurysms

Coiling introduced a paradigm shift in cerebral aneurysm treatment as it provided a means of occluding the aneurysm sac with no surgical intervention (Guglielmi et al., 1991). One of the earliest studies of thrombus formation following coiling examined 17 aneurysms removed at autopsy and one removed after surgery (Bavinzski et al., 1999). It was found that full occlusion observed at initial angiography did not necessarily translate into full occlusion at gross pathological examination following excision. The study also found that unorganized thrombosis or incomplete clot maturation, i.e., replacement of the clot with fibrous tissue, could later lead to aneurysm growth and rupture. The International Study of Unruptured Aneurysm Investigators then found that for 451 cases, 55% had full occlusion, 24% were partially occluded and 18% had no occlusion (Wiebers et al., 2003). A review examined case-fatality, morbidity and effectiveness in preventing bleeding by enrolling 1,379 patients with at least 1,621 aneurysms (Lanterna et al., 2004). It was found that none of the aneurysms with full occlusion bled after embolization with coils. For partially occluded aneurysms, bleeding was observed in those greater than 10 mm in size. A meta-analysis of 71 studies enrolled 5,044 patients with 5,771 aneurysms (Naggara et al., 2010). One aim of this study was to estimate treatment efficacy using immediate and follow-up results. Immediately after treatment, it was found that 86.1% of aneurysms had full occlusion or a neck remnant only, 10.3% of aneurysms were partially occluded and that coil treatment was unsuccessful in 3.6% of cases. During follow-up, recanalization of the aneurysm sac was observed in 24.4% of patients over a 0.4–3.2 year period. Finally, the Analysis of Treatment by Endovascular Approach of Non-ruptured Aneurysms (ATENA) study enrolled 649 patients with 1,100 aneurysms (Pierot et al., 2008). It was found that 98.4% were treated with coils alone, while a few cases required additional techniques, such as stent-assisted coiling. Following intervention with coils only, 59.0% of aneurysms had full occlusion, 21.7% had a neck remnant and 19.3% were partially occluded. The studies demonstrated that coil embolization was an effective mode of treatment for a wide range of aneurysm cases, but that sometimes, additional techniques were required. They also demonstrated that full occlusion could be achieved using coils, and that usually, this led to stability of the aneurysm sac. A summary of these studies is presented in Table 1.

**Table 1:** Summary of occlusion outcomes for coiling studies.

**Study**	**Number of aneurysms**	**Full occlusion (%)**	**Partial occlusion (%)**	**No occlusion (%)**	**Other findings**
Bavinzski et al.	18	–	–	–	Full occlusion at initial angiography did not necessarily translate to longer term
Wiebers et al.	451	55	24	18	Status unknown in 3% of cases
Lanterna et al.	1,621				Full occlusion with coils prevents rebleeding
Naggara et al.	5,771	86.1	10.3	3.6	Recanalization observed in 24.4% of cases
Pierot et al.	1,100	59.0	19.3	–	Neck remnant in 21.7%

### Flow diversion in unruptured aneurysms

In recent years, flow diverters have gained popularity in treating large, giant or traditionally untreatable aneurysms (Szikora et al., 2010). The main principle of flow diverters is that they alter the local haemodynamics by redirecting flow along the main vessel and reducing flow into the aneurysm sac (Kulcsár et al., 2011). The reduced flow in the aneurysm sac often results in the formation of an occlusive clot. The Pipeline for Uncoilable or Failed Aneurysms Trial established the efficacy and safety of the Pipeline endovascular device (Becske et al., 2013). The study enrolled 108 patients with giant and wide-necked aneurysms. It was found that flow diverters were an effective means of treating aneurysms considered difficult to treat using other methods. After 180 days, it was found that 73.6% of aneurysms were occluded. One year after treatment, 86.8% of aneurysms had full occlusion, 5.5% had a neck remnant, 5.5% were partially occluded and 2.2% had no occlusion. Adverse neurological events were relatively low (5.6%). A different study published in the same year established safety and efficacy in Pipeline endovascular devices and Silk stents (Piano et al., 2013). The study enrolled 101 unruptured aneurysms. Six months after treatment, it was found that 86% of aneurysms had full occlusion. Of the original cohort, 53 were evaluated 1 year after intervention. It was found that none of the aneurysms showed recanalization and complete shrinkage of the aneurysm sac was evident in 61% of aneurysms which could be assessed. Mortality and morbidity rates were both found to be 3%. As these two studies carried out work to establish the efficacy and safety of flow diverter placement, various other hospitals and medical centers in high income countries were increasingly convinced of flow diversion as a viable alternative and wanted to better understand the impact of flow diverter use. A meta-analysis of 29 studies enrolled 1654 aneurysms and found complete occlusion in 76% of cases (Brinjikji et al., 2013). When considering aneurysm size, complete occlusion was observed in 80% of small aneurysms, 74% of large aneurysms and 76% of giant aneurysms. Morbidity (5%) and mortality (4%) were not negligible. The uncertainty about the adverse neurological outcomes led to a retrospective study in 906 aneurysms (Kallmes et al., 2015). It was found that neurologic morbidity and mortality following placement was 8.4%, while spontaneous rupture rate was 0.6%. Ischemic stroke and intracranial hemorrhage were 2.4% and 4.7%, respectively. In-stent stenosis was observed in 0.3% of cases. Finally, a systematic review of 18 studies with 1704 aneurysms found that average follow-up time was 9 months (Briganti et al., 2015). At the point defined as “final follow-up,” complete aneurysm occlusion was observed in 81.5% of cases. The studies relating to flow diversion demonstrated that flow diverters were relatively safe and effective for treating cerebral aneurysms which were difficult to treat with other endovascular approaches. The occlusion rates following flow diverter placement were generally high and those aneurysms not fully occluded postoperatively were often more fully thrombosed in follow-up observations. The risk of neurologic complication following flow diverter placement highlighted the importance of determining when to make use of this mode of treatment. A summary of these studies is presented in Table 2.

**Table 2:** Summary of occlusion outcomes for flow diversion studies.

**Study**	**Number of aneurysms**	**Full occlusion (%)**	**Partial occlusion (%)**	**No occlusion (%)**	**Other findings**
Beckse et al.	108	86.6	5.5	2.2	Neck remnant in 5.5%. Adverse neurological events in 5.6%.
Piano et al.	101	86	–	–	Recanalization and shrinkage of sac in 61%. Mortality and morbidity rates 3%.
Brinjikji et al.	1,654	76	–	–	80% in small aneurysms. 74% in large aneurysms. 76% in giant aneurysms.
Kallmes et al.	906	–	–	–	Lowest complication rates observed in small aneurysms
Briganti et al.	1,704	81.5	–	–	

As evidenced by the studies discussed in the last two subsections, much of the reporting of clotting outcome is inferred from the extent of occlusion in the aneurysm sac. This is relatively easy to observe at the end of a procedure as angiography is already used during intervention (Bederson et al., 2000; Byrne et al., 2010; Thompson et al., 2015). Even though the extent of occlusion is thought to indicate re-rupture risk, especially in ruptured aneurysms, the main challenge with inferring clotting success from such an indirect measure is that the nature, maturity and stability of the clot cannot be accounted for (Johnston et al., 2008). This is, therefore, a very limited assessment when judging the success of devices as two devices may have similar occlusion patterns at a given time point, but ultimately lead to very different thrombus characteristics (Reul et al., 1997; Böcher-Schwarz et al., 2002). An *in vitro* study that evaluated flow-diverter induced thrombosis showed that even when occlusion patterns were similar for two flow-diverters with different porosities, the microscopic composition of the clots was not identical (Gester et al., 2016). The long term stability of the clot is also influenced by factors not limited to occlusion. Various studies have shown that thrombus organization and endothelialisation are more important predictors of long-term outcome following intervention and occlusion (Bavinzski et al., 1999; Kadirvel et al., 2014; Szikora et al., 2015).

Even with the insights gained from the above-mentioned studies, a number of clinical questions relating to endovascular intervention remain unanswered. The main question that has driven much of the effort directed towards developing computational interventional planning tools is when is an unruptured cerebral aneurysm likely to rupture and therefore, when should it be treated? Given that the outcome of intervention is embolization of the aneurysm sac and that this is thought to influence rupture risk, many tools are now attempting to answer questions relating to clot growth following device placement. Ideally, such models should predict immediate and long-term clotting outcome and stability. They should also enable optimization of anticoagulant regimens, to avoid undesirable spontaneous clotting and encourage aneurysm sac embolization initiated by device placement.

## Demands on computational models of thrombosis in cerebral aneurysms for interventional planning

For computational models of cerebral aneurysm thrombosis to be an effective tool in predicting occlusion for interventional planning purposes, a few considerations must be made. The models developed must be sophisticated enough to capture salient details of the occlusion process. They also need to be simple enough so they can provide information to a clinician within a reasonable timeframe that is compatible with clinical routine. In this section, the requirements of interventional planning and optimization of anticoagulant regimens for endovascular treatment of cerebral aneurysm are considered. A discussion of the key outputs from the model, level of computational burden admissible based on clinical constraints and level of detail that the model must account for is presented. This then provides a basis of judgment when considering different thrombosis modeling techniques and their suitability for interventional planning.

The key outputs from a computational model of thrombosis for interventional planning would be an indication of the extent to which the aneurysm sac is occluded following intervention, and the time from occlusion to clot maturation. Even though its focus was on ruptured aneurysms, the CARAT study quantified risk of rerupture based on occlusion, highlighting the importance of this factor (Johnston et al., 2008). It was found that rupture risk was 1.1% for full occlusion, 2.9% for 91–99% occlusion, 5.9% for 70–90% occlusion and 17.6% for <70% occlusion. Various other studies focusing on unruptured aneurysms have also highlighted the importance of occlusion outcome following device placement (Byrne et al., 2010; Saatci et al., 2012; Brinjikji et al., 2013). For coil embolization, occlusion outcome is evident immediately after intervention (Pierot et al., 2008). With flow diverters, occlusion is typically measured over several months following intervention (Briganti et al., 2015). While occlusion of the sac is an important clinical endpoint following intervention, endothelialisation and thrombus organization are thought to indicate the long-term stability of the clot and the aneurysm (Bavinzski et al., 1999; Kadirvel et al., 2014). Predicting immediate and long-term outcome of clotting would provide insight into the nature and eventual maturity of the clot, and the time between occlusion and clot maturity. Such comprehensive information would be beneficial when selecting a device and when optimizing adjuvant anticoagulant regimen. In addition, information about the progression from occlusion to maturity would provide insight into this critical period that determines clot stability, and enable the clinician to make adjustments that would minimize the risk of clot dissolution. The ability to model such a system computationally would require a well-resolved, multiscale approach, which could bridge the widely varying timescales involved. In the flow diverter case, for example, occlusion may happen within a matter of minutes while clot maturation may take 6 months (Szikora et al., 2010; Tähtinen et al., 2012; Heller et al., 2013). The ability to accommodate both processes within a single framework would be beneficial.

In a clinical setting, turnaround time is an important factor when considering the applicability of different medical interventions. Computational models should be designed so the inputs required can be aligned to existing systems with relative ease and the outputs can be obtained within reasonable time. An important input for a computational model of thrombosis would be magnetic resonance imaging (MRI) or computerized tomography (CT) scans from which the patient's aneurysm can be reconstructed. Even though some studies have shown that three-dimensional rotational angiography (3DRA) provides higher anatomical resolution, this modality of imaging would subject the patient to catheterization during the planning phase (Geers et al., 2011; Ren et al., 2016). Other key inputs would include an indication of the pharmacological status and clotting profile of the patient, and flow rates in the blood vessel of interest. A study examining the time taken for an intracranial aneurysm to be coiled found that on average, the mean procedural time from the first diagnostic angiographic run to the last angiographic run after embolization was 57.3 min (De Gast et al., 2008). Adding a computational model for interventional planning purposes would increase the procedural time. For unruptured aneurysms with no imminent risk of rupture, adding a day or two to the procedure would be admissible, given the value of the information that such a model would provide. For unruptured aneurysms with imminent risk of rupture, procedural time made lengthier by predictive computational tools could prove fatal for the patient. The ideal computational model would provide valuable information within as short a period of time as possible and minimize the time added to the overall procedural time. The two key determinants of the level of complexity in the model are the biochemistry and the local fluid dynamics, and these are discussed in the two paragraphs that follow.

One of the key inputs for the computational model is the clotting profile of the patient, representing the biochemical aspect of coagulation. The ideal input would be a specific patient's clotting profile derived from a clinical coagulation test. Hemker et al. have demonstrated how this can be achieved by developing a technique that tracks the change in fluorescence of a fluorogenic thrombin substrate during clot formation in plasma (Hemker et al., 2000, 2002; Wagenvoord et al., 2006; Hemker and Kremers, 2013). This signal is then translated into a thrombin generation curve. The methodology can account for hypo- and hypercoagulability. Many clotting models have been presented in literature, with some including all the equations that describe the clotting process and others presenting a reduced form of the coagulation process (Hockin et al., 2002; Panteleev et al., 2006; Wagenvoord et al., 2006; Anand et al., 2008; Filipovic et al., 2008; Purvis et al., 2008; Xu et al., 2008; Leiderman and Fogelson, 2011; Storti and Vosse, 2014). The ideal model would incorporate the minimum number of equations for capturing a unique patient's clotting profile. To further simplify the biochemical complexity in the model, patients could be categorized as hemophiliac, normal or pro-thrombotic, and this could then reflect in the model through adjustment of various protein concentrations and/or clotting equations. Another key aspect that would need to be incorporated in the model is the effect of anticoagulant regimens on clotting outcome. Given that the administration of anticoagulant and antiplatelet therapy is a requirement for endovascular procedures, the effect of these drugs on the clotting process must be accounted for and the computational model could be a tool for optimizing these regimens (Faught et al., 2014).

The other key input is the local hemodynamics in the region of clot formation. This is largely influenced by the cerebral aneurysm geometry, the boundary conditions and the physical properties of blood. The geometry used to simulate the clotting process is quite important in predicting occlusion outcome. Using idealized geometries is too simple to capture the features unique to specific patients. Over time, a large enough database of patient cases may be built up so generalized geometries could be generated from this pool, and these could then give an accurate enough prediction of clotting outcome. Currently, patient-specific geometries are still an important part of the process. A specific patient's cerebral aneurysm geometry can be derived from medical images obtained during the diagnostic stage. Through reconstruction techniques, the scans can be prepared for computational models (Villa-Uriol et al., 2011). The boundary conditions applied to the model are a potential area for simplification. One study has shown that for computational fluid dynamics (CFD) based rupture predictions, the effect of flow variability is an important factor to consider and efforts were made to model boundary conditions more stochastically to account for uncertainty and variability (Sarrami-Foroushani et al., 2016). Another potential area for simplification relates to the physical properties assigned to blood. The properties of clotted blood differ from those of flowing blood (Diamond, 1999; Muthard and Diamond, 2012). This needs to be accounted for in a computational model as regions of clotted blood are likely to affect the rate at which coagulation proteins are delivered to the clot site, and the rate at which they react with one another. Various approaches can be taken to account for this difference in physical properties and each will have a different computational cost (Bodnár and Sequeira, 2008; Leiderman and Fogelson, 2011; Storti and Vosse, 2014). These are discussed in the section that follows.

## Computational models of thrombosis and aneurysm-specific computational models of thrombosis

Various computational models of physiological and pathological clotting have been developed and presented in literature. These typically consist of different physical subsystems (e.g., biochemical reactions, platelets, hemodynamics) that are coupled together to simulate the clotting process. These subsystems are referred to as modules in this discussion. Most models are first developed from a physiological clotting basis which can be validated and verified. Some of the existing physiological models could be adapted and developed for computational models of cerebral aneurysm thrombosis. In this section, we present existing models found in literature. We begin with general physiological models and then discuss those designed specifically for cerebral aneurysm thrombosis.

### General physiological models

Initially, computational models of the clotting process were developed to improve understanding of the hemostatic system. Some models provided a means of coupling various complex modules and of determining the influence of these subsystems on the whole system. In this subsection, the different models are described and grouped according to their foci, namely, coagulation network models, platelet models, reaction-mass transport models and integrated models. This is then followed by a comparison of the different models and their applicability to patient-specific modeling in cerebral aneurysms.

#### Coagulation network models

Coagulation network models focus solely on the reactions which result in the formation of thrombin and ultimately, fibrin. These models are of interest when developing a computational model as they provide an accessible means of understanding how to model chemical reactions numerically.

In their work, Hemker et al., Hemker and Kremers, and Wagenvoord et al. demonstrate a technique for obtaining a thrombin generation curve from a coagulation test (Hemker et al., 2000, 2002; Wagenvoord et al., 2006; Hemker and Kremers, 2013). During clot formation in plasma, the change in fluorescence of a fluorogenic thrombin substrate is monitored, and this signal is converted to a thrombin generation curve. An analytical formula that describes the thrombin generation curve is presented. Three variables are required to generate a curve from this formula. These can be obtained from a database of normal clotting profiles or from an individual patient during testing. The model presented here would apply to a clinical setting as the parameters for any patient can be determined with relative ease. In addition, the model simplifies the computational cost of incorporating biochemistry.

Butenas et al., Hockin et al., Jones and Mann, and Mann et al. patented a computer programme that can determine the efficacy of blood clotting agents (Jones and Mann, 1994; Butenas et al., 1999; Hockin et al., 2002; Mann et al., 2006). The model is based on a comprehensive network of the coagulation cascade and accounts for all the reactions that occur during the clotting process. It comprises 27 reactions and 42 reaction constants, and is based on experimental observations. This model accounts for most of the initiation reactions linked to tissue factor, the propagation phase and for several inhibitory reactions. The reactions are expressed as time-dependent partial differential equations and are computed using a fourth-order Runge-Kutta solver. This model is the gold standard of comprehensive computational coagulation cascade models. It would, however, prove difficult to determine the parameters required for this model on a per patient basis, in a clinical context.

#### Platelet models

The key role played by platelets in coagulation is the central feature of platelet-centric models. Such models place focus on the adhesion, activation, accumulation and aggregation of platelets, and the coagulation reactions supported by the platelet membranes.

Filipovic et al. developed a two-dimensional model that considered the interactions between plasma and platelets using a dissipative particle dynamics (DPD) method (Filipovic et al., 2008). Blood is considered a colloidal mixture where platelets and plasma are modeled as mesoscale particles. The movement of particles is described by Newton's second law and unlike the more traditional continuum methods, greater prominence is given to the contribution of individual particles. These particles are sufficiently small to track the motion of plasma and platelets, but are larger than atoms, hence molecular dynamics approaches were unnecessary for resolving changes in the colloidal system. The interaction between the particles is described by conservative (repulsive), dissipative and random (Brownian) forces, and the model accounts for both active and inactive platelets. The DPD differential equations are integrated using a velocity Verlet algorithm. At the end of each time step, the particle's position is calculated using the Euler forward method, while the velocity and forces are calculated using a mid-step velocity. Platelet-wall adhesion forces are also included in the framework. This is achieved by incorporating an attraction (bonding) force, which is modeled as a linear spring attached to the platelet surface on the one end, and to the wall or an adhered platelet on the other. An additional parameter relating to the wall domain, which supports these forces, is also included. The thrombus was seen to grow as more platelets accumulated, were activated and adhered to the thrombus mass.

Chatterjee et al. and Purvis et al. presented a model that can predict all the events that take place during platelet signaling and includes the activity of both resting and activated platelets (Purvis et al., 2008; Chatterjee et al., 2010). The model accounts for molecular mechanisms that accurately describe platelet homeostasis and response to adenosine diphosphate (ADP), which activates platelets. Seventy-seven reactions and seventy species were used to describe the behavior of the human platelet. Even though the reactions were not exhaustive, the model accurately reproduced key features of platelet activity, including intracellular calcium activity. The computational model was developed from experimental observations, where high resolution electron micrographs were used to examine intracellular calcium concentration in platelets following the addition of ADP. The reactions in the computational model are described by ordinary differential equations and are solved in the Systems Biology Toolbox in MATLAB. Flamm et al. developed this model further by including the influence of flow and enabling the prediction of patient-specific platelet network responses (Flamm et al., 2012). This two-dimensional multiscale model consists of four submodules. The Lattice Boltzmann submodule accounts for the flow of the fluid in the solution domain. The finite element method submodule computes the convection-diffusion-reaction equations, and accounts for the changing concentrations of ADP and thromboxane. For this submodule, the Crank-Nicholson scheme was used for the transient term. The lattice kinetic Monte Carlo submodule accounts for motion of individual platelets in the fluid and for platelet interactions with the surface. Appropriate time steps and event probability are determined using the Next Reaction Method. Finally, a neural network model submodule is used to simulate platelet calcium signaling and this specific module was customized for each individual donor.

Several platelet models place emphasis not only on platelet activation, but also on the adhesion of platelets to other platelets, and their interactions with the vessel wall. Mody and King produced a multiscale, three-dimensional model of platelet adhesion dynamics (Mody and King, 2008a,b). They realized that much of the existing literature on particulate flows focused on spherical particles. These models were somewhat inappropriate for modeling platelet interactions, as active platelets exhibit filopodia on their surface and inactive platelets resemble flattened ellipsoids. In their model, hydrodynamic calculations are used to account for the behavior of platelets, modeled as oblate spheroids, in a linear shear flow regime. The model couples two distinct modules. The first module calculates the forces (gravity, bond, repulsion) acting on individual particles in the fluid. Special attention is paid to repulsive forces at close range and their inclusion in the model can account for the interaction of the platelet with the wall. The second module then computes the motion of the particles in the fluid, based on the forces acting on the particle, the external fluid field and rigid body motion of the particle. This is achieved by numerically solving the equations of fluid mechanics relating to flows within the Stokes regime. The integral form of the Stokes equation is solved using the completed double layer-boundary integral equation method (CDL-BIEM), a boundary elements method. To validate the model, collision efficiency calculated using the model was compared to experimentally-determined efficiency.

Wu et al. developed Mody and King's approach by modeling the platelets as deformable particles (Mody and King, 2008a,b; Wu et al., 2014). The three-dimensional model comprises three coupled submodules, which account for the elastic properties of the platelet, flow in the fluid domain and platelet adhesion kinetics. The platelet elasticity submodule incorporates the behavior of the cell membrane and the cytoskeleton, and the flow submodule uses the Lattice Boltzmann method to account for blood flow. Finally, the adhesion module which accounts for platelet-platelet adhesion and platelet-vessel wall interactions is accounted for by a kinetics-based stochastic model. Immersed boundary methods are used to couple the flow and the platelets, with the fluid described by an Eulerian approach and the platelets by a Lagrangian approach. Good agreement was found between the model's results and experimental studies carried out by Mody et al. (2005).

#### Reaction-mass transport models

Reaction-mass transport models account for coagulation reactions and the transport of coagulation proteins in the flowing bloodstream. This enables the investigation of the effect of convection and/or diffusion on these reactions. Given the key role played by local hemodynamics in clotting outcome in aneurysms, these models provide a good basis for understanding how best to couple biochemical reactions with local hemodynamics.

In the early nineties, Basmadjian presented a model for examining the link between flow and chemical reactions (Basmadjian, 1990). This is achieved by modeling a single, generic, reaction event at the blood vessel wall. The model translates *in vitro* observations of coagulation into mathematical expressions without oversimplifying the mathematics or biological events. At the vessel wall, reactions can be modeled as algebraic equations under steady state conditions and as ordinary differential equations under transient conditions. The size of the vessel influences the assumptions which can be made. For vessels with diameter greater than 0.1 mm, radial changes in the transport resistance and protein concentration are confined to the boundary layer. Boundary conditions can therefore account for this variation. For vessels with diameter less than 0.1 mm, that assumption does not hold because the boundary layer is not thin, relative to the rest of the flow field. Even though this model is limited to the development of mathematical equations, it provides good insight into convection-diffusion-reaction equations and is beneficial for developing a computational model from first principles.

Panteleev et al. developed mathematical formulae of surface and volume reactions based on *in vitro* studies and observations (Panteleev et al., 2006). The model consists of 27 reactions and fifty kinetic constants, and describes a reaction-diffusion system. The computational framework accounts for the different membranes on which reactions take place (TF-bearing cells and platelets), and implements updates to protein concentrations in a biphasic manner. The concentrations of proteins found on endothelial cells are calculated using surface densities, while those found on platelet membranes are updated using volume concentrations. The two phases are linked by boundary conditions. The differential equations are solved using the Runge-Kutta-Fehlberg method of the second (third) order written in a C/C++ compiler.

Bodnar and Sequeira presented a convection-diffusion-reaction model of coagulation (Bodnár and Sequeira, 2008). Their computational framework is based on a mathematical model developed by Anand et al. whose work is presented in the “*Integrated Models*” section (Anand et al., 2006, 2008). The model is implemented in a three-dimensional cylinder and the coagulation process is described by 23 coupled equations that account for reactions and mass transport. A finite volume solver is used for spatial discretization and integration is achieved through the use of cell-centered fluxes. An explicit Runge-Kutta formulation is used for time integration and pressure stabilization is achieved through the use of an artificial compressibility approach. The clot and fluid regions are differentiated by viscosity, which increases linearly with fibrin concentration. Above a certain fibrin threshold, the viscosity takes its upper limit value, clearly delineating the clot region. The upper limit viscosity value is the viscosity of blood multiplied by a factor of 100. The variation of viscosity is one approach to accounting for the physical differences between plasma and clotted blood. In some respects, the model simplifies the one presented by Anand et al. by considering blood to be a generalized Newtonian fluid with shear-thinning properties rather than a viscoelastic material. In addition, platelet reactions are not modeled directly.

#### Integrated models

Integrated models consider the different aspects of clotting. Often, they incorporate coagulation reactions, platelet activity and hemodynamics. The benefit of these models is they provide a mechanism for coupling the different systems that contribute to the clotting process and are therefore more readily applicable to interventional planning models. There are pathological cases where certain aspects of the clotting process may require alteration, but it is often easier to alter or remove modules than to develop coupling approaches to include new modules. The choice of an appropriate integrated model for interventional planning will depend on the emphasis of that model and on the computational efficiency of the numerical approach.

Sorenson et al. produced a two-dimensional computational model of platelet deposition and activation in flowing blood, with the intention of modeling clot development on biomaterials (Sorensen et al., 1999a,b). The model accounts for platelet-platelet and platelet-vessel wall adhesion. The model also incorporates the role of biochemistry by including ADP, thromboxane, prothrombin, thrombin and antithrombin. The changes in the concentrations of the biochemical species and platelets are described and computed by convection-diffusion-reaction equations. Fluid flow is described by the Navier-Stokes equations and contributes to the transport of the different chemical species. First-order, low-numerical-diffusion-fluctuation splitting schemes are used to compute the convection terms and the coupled system is solved using a segregated iterative procedure. This procedure requires convergence for each equation and for the entire system. Temporal evolution is achieved in two steps. The first computes a steady-state solution in pseudo-time. Advancement of this solution in real-time is realized in the second step. To validate the computational model, comparisons are made with experiments where blood flows over collagen in a parallel plate configuration. It was found that four parameters were necessary for fitting the computational model to experimental results. These parameters are shear-dependent platelet diffusivity and three reaction rate constants dependent on platelet deposition.

In their mathematical model, Anand et al. tracked the growth of a clot from initiation to lysis (Anand et al., 2006). The model accounts for coagulation reactions, platelet activity and haemodynamics. The biochemical changes that occur during clotting are described by 23 convection-diffusion-reaction equations developed from experimental observations. The initial concentrations of the proteins are those in human plasma. It is assumed that 0.1% of activated enzymes are present in the blood. The activity of resting and activated platelets is also accounted for, with some constants being taken from literature. The activation of platelets depends on concentration and on mechanical variables in the flow domain. Blood is modeled as a viscoelastic fluid with shear-thinning properties and the constitutive equations for the fluid and clot regions are similar. The boundary between clot and fluid can be tracked, making it possible to apply different material properties, such as viscosity, to different regions. This enables assignment of different transport properties to proteins in the clot region, a highly desirable feature in an interventional planning tool. In a another paper, the same authors consider the case where blood is static (Anand et al., 2008). This reduced the complexity of the model by considering a diffusion-reaction system rather than a convection-diffusion-reaction system, and enabled validation with experimental results of pooled blood. The equations are solved using MATLAB'S partial differential equation solver. In this model, clot growth is observed in areas where fibrin concentration exceeds a specified threshold (350 nM) and fibrinolysis takes place where the concentration drops below this threshold.

Leiderman et al. and Leiderman and Fogelson presented a two-dimensional computational model accounting for coagulation reactions, platelet activity and haemodynamics (Leiderman et al., 2008; Leiderman and Fogelson, 2011, 2013). The Navier-Stokes equations describe the flow conditions and the clot region assumes a different porosity value to plasma. A fractional-step projection method is used to solve these equations. The evolution of coagulation protein concentrations are described using partial differential equations, and distinction is made between those that are found on platelet membranes, on the subendothelial layers and those circulating in the bloodstream. In addition, the model accounts for the altered transport properties of proteins moving within the clot. Various states of platelet activation are considered and the molecules which contribute to platelet activation are represented. The convection of proteins and platelets is solved using LeVeque's convection algorithm, while diffusion is solved using spatial-differencing approximation and Crank-Nicholson time discretization (LeVeque, 1996). A second-order Runge-Kutta solver is used to calculate the changes in species concentration.

In their two-dimensional model, Xu et al. considered the spatial scales that contribute to the coagulation process (Xu et al., 2008, 2009a,b). Platelets are microscalar objects and are modeled using the Cellular Potts method. In this method, the motion of a platelet is governed by the effective energy of the cell, which can be calculated using the Metropolis dynamics algorithm. This module also accounts for platelet-platelet and platelet-vessel wall interactions, and for platelet aggregation, adhesion and activation. Platelet activation occurs once a specified thrombin threshold is exceeded. The macroscale continuum level accounts for blood flow, which is described using the Navier Stokes equations, and for the transport of coagulation proteins. The biochemical reactions are based on the model developed by Butenas et al. and are described using partial differential equations (Butenas et al., 1999). An interface module allows for smooth transmission of information between the two scales, which are spatially superimposed. In order to track the interface between the growing clot and plasma, an algorithm similar to the volume of fluid method is used. In their updated model, the same authors implement the model in the venous circulation and treat the clot as a porous medium (Xu et al., 2010). The role played by platelet membranes is further developed and each individual platelet's contribution to thrombin production is accounted for.

A two-dimensional hybrid dissipative particle dynamics-partial differential equation (DPD-PDE) model was presented by Tosenberger et al. (2013). Motion is described by Newton's second law and accounts for conservative, dissipative and random forces between particles. This equation is integrated using the forward Euler or modified velocity-Verlet algorithm. Platelets and plasma are modeled using dissipative particle dynamics. Given that DPD focuses on the mesoscale, each particle represents a volume of matter rather than an individual molecule. The radius of all the particles was chosen to match that of platelets. The changes in fibrin concentration are accounted for by a partial differential convection-diffusion-reaction equation. A finite-difference Alternating Direction Implicit method is used to compute this partial differential equation and the convection term is treated with a first order upwind scheme. The model accounts for the changes that occur during clot growth and makes a distinction between strongly- and weakly-bound platelets in the clot mass. In addition, the model differentiates between platelets covered by fibrin and platelets not covered by fibrin. This distinction is important as the latter group cannot attach new platelets.

In their two-dimensional model, Storti et al., and Storti and Vosse focused on fluid-structure interaction between the growing platelet mass and blood flow (Storti and Vosse, 2014; Storti et al., 2014). This model accounts for platelets, the flow field, biochemical reactions and the mechanical response of the growing clot. The biochemical model is based on Sorenson et al.'s framework, which includes active and inactive platelets, ADP, thromboxane, prothrombin, thrombin and antithrombin (Sorensen et al., 1999a). The changes in the quantities of these variables are calculated by a convection-diffusion-reaction equation. Robin boundary conditions are applied to the injured part of the vessel, Neumann to the healthy and Dirichlet to the inlet. One of the key features is a platelet plug growth model, which is based on the concentration of platelets bound to the site of injury. This includes platelets bound directly to the vessel wall, and those bound to other platelets. An Arbitrary Lagrangian Eulerian method is used to account for the changing mesh as growth occurs. The interface of the platelet plug is clearly defined and the plug is a deformable solid which responds to local haemodynamic changes, which are described by the Navier-Stokes equations. The non-linear term in the equations is treated explicitly. A robust fluid-structure solver enables calculations of the interaction between the platelet plug and local haemodynamics. This solver operates in a sequential fashion, where the fluid forces acting on the solid plug are first calculated and then deformations resulting from the forces are imposed on the fluid field. This calculation is carried out at every time step and an implicit Euler method is applied.

Stalker et al. Tomaiuolo et al. and Welsh et al. employed a systems approach to elucidate the mechanisms at play during clot formation in arterioles and venules (Stalker et al., 2014; Tomaiuolo et al., 2014; Welsh et al., 2014, 2016). Computational and experimental techniques were used to examine the microenvironment of the clot. Experimental observations showed that the clot consisted of a core, where platelets were tightly packed, and a shell, which allowed for greater diffusion of molecules. Two-dimensional computational models were developed to examine molecular transport within this complex structure. In the first model, platelets are modeled explicitly and are represented as ellipses. This model was used to examine the effect of packing density and thrombus size on blood flow velocity. In the second model, the clot is modeled as a porous medium consisting of two regions. This model also accounts for species transport, which is described using a convection-diffusion-reaction equation. Flow in the vessel is described by Stokes' equation and flow in the clot by a Brinkman equation. To account for the different flow properties in the core and shell regions, different porosity, permeability and effective diffusion coefficient values are assigned.

#### Comparison of the different approaches and applicability to computational models of cerebral aneurysm thrombosis

The biochemical reactions that contribute to the clotting process are crucial for the formation of fibrin and stabilization of the platelet mass. Without these reactions, the clotting process is severely hampered. In developing a computational model of thrombosis in cerebral aneurysms, it is imperative that these reactions are included in some form of a biochemistry module. The main challenge lies in selecting a model that can account for sufficient detail, while being simple enough for parameters to be determined within a clinically relevant timeframe. The work presented by Hemker et al., Hemker and Kremers, and Wagenvoord et al. would be ideal for a cerebral aneurysm thrombosis model, as it presents a link between a unique patient's thrombin curve and an analytical description which could be used in the computational model (Hemker et al., 2000, 2002; Wagenvoord et al., 2006; Hemker and Kremers, 2013). This can be determined in a clinical test and translated to the model with relative ease. While the model presented by Hockin et al. and Mann et al. is a good representation of reality, it contains far too many rate constants for use in clinic, on a per patient basis (Hockin et al., 2002; Mann et al., 2006). This model would be more applicable if one were to consider general population characteristics which could be pre-programmed, for example, normal subjects, hemophilia subjects and thrombotic subjects. Either model could be used for the biochemistry module in a cerebral aneurysm thrombosis model. If a model were to be developed for this specific purpose, it would combine the simple link between the thrombin curve and a specific patient's profile, and the rigor of a model which represents physical reality. In addition, an improved understanding of cerebral aneurysm thrombosis biochemistry would enable the development of a coagulation network that is unique to the pathology.

Including platelets in an interventional planning model would be beneficial, as it would enable optimization of antiplatelet therapy administered alongside endovascular device placement. Several approaches can be taken to include such a module. The model presented by Chatterjee et al., Flamm et al. and Purvis et al. is comprehensive in describing platelet signaling and changes that take place during platelet activation (Purvis et al., 2008; Chatterjee et al., 2010; Flamm et al., 2012). A sensitivity study could be carried out to determine the key reactions, incorporating some of the salient features into an interventional planning framework. This model is attractive as it already accounts for patient specificity and could therefore be used for optimization of antiplatelet therapy with relative ease. Filipovic et al. couple platelet activity to mass transport through DPD (Filipovic et al., 2008). The detailed modeling of platelet behavior is perhaps excessive for a cerebral aneurysm thrombosis model. The deviation from the usual Navier-Stokes equations approach does, however, demonstrate a viable alternative when considering seamless coupling between platelet behavior and flow. The models presented by Wu et al. and Mody and King focus on the interactions between individual platelets and the interaction between platelets and the vessel wall (Mody and King, 2008a,b; Wu et al., 2014). While these models accurately capture salient interactions, they could be over-developed for a cerebral aneurysm thrombosis model. Given the strong need for feedback within clinically relevant timeframes, including individual platelet interactions is likely to increase overall computation time for minimum gain in the final clotting result.

Reaction-mass transport models are a good alternative to integrated models. They clearly demonstrate how to couple the crucial aspects of thrombosis, i.e., local hemodynamics and reactions (platelets or biochemical). In developing a cerebral aneurysm thrombosis model, the coupling approaches could either replicate the models, or they could couple a hemodynamic method to the biochemical reaction or platelet models described in the previous two paragraphs. Basmadjian, Panteleev et al., and Bodnar and Sequeira focus on coupling biochemical reactions to flow (Basmadjian, 1990; Panteleev et al., 2006; Bodnár and Sequeira, 2008). The model presented by Basmadjian is helpful when developing an understanding of how best to implement boundary conditions in the context of flow. In their reaction-diffusion model, Panteleev et al. present a further level of sophistication by distinguishing between biochemical activity at the vessel wall and activity in blood. Bodnar and Sequeira present a convection-diffusion-reaction model that can account for the effect clot on the flow field. This last model could be applied to a cerebral aneurysm thrombosis model as mass transport is already coupled to reactions and the numerical approaches have relatively low computational cost.

Integrated models have robust mechanisms for coupling different modules. One of the key tasks is determining whether it is necessary that all these modules are included in the model. The other major challenge with all the existing integrated models is that their efficacy has been demonstrated in two-dimensional geometries. Cerebral aneurysm geometries are often complex, three-dimensional structures and the transferability of methods from one dimension to the other would need to be examined. Sorenson et al. developed one of the earliest integrated models, which extracted the key equations for platelet activation and thrombin formation, and coupled these to flow (Sorensen et al., 1999a,b). The biochemistry/platelet module in this study would apply to a cerebral aneurysm thrombosis model as it identifies the key equations and specifies that four parameters are needed to fit experimental results to computations. These could be determined with relative ease in a clinical setting, on a per patient basis. The mathematical model presented by Anand et al. added a level of sophistication by altering viscosity to account for the effect of the growing clot on the flow field (Anand et al., 2006, 2008). The work presented by Bodnar and Sequeira, discussed under the reaction-mass transport section, implemented a simpler version of Anand et al.'s work computationally, demonstrating its efficacy in a three-dimensional geometry (Bodnár and Sequeira, 2008). Rather than using viscosity, Leiderman et al. and Leiderman and Fogelson used porosity to account for the effect of the growing clot in their model (Leiderman et al., 2008; Leiderman and Fogelson, 2011, 2013). In addition, their model accounts for the altered transport of biochemical species in the clot region and for the various states of platelet activation. The models presented by Sorenson et al. Anand et al. and Leiderman and Fogelson rely on convection-diffusion-reaction equations for coupling various entities. Xu et al. take a slightly different approach by spatially superimposing a microscale platelet model, based on the Cellular Potts method, with a macroscale flow model (Xu et al., 2008, 2009a,b, 2010). In addition, they use the volume of fluid method to carefully track the surface of the growing clot, making it possible to assign different porosity values to the region. A combination of Sorenson et al.'s biochemistry module with Anand et al. or Leiderman et al.'s method for accounting for the effect of the growing clot on the flow field would apply to a cerebral aneurysm thrombosis model. Given the complexity of the aneurysm geometry, the ability to track the surface of the growing clot in a cerebral aneurysm thrombosis model is important and the method presented by Xu could be of benefit. In a similar fashion to Filipovic et al.'s model, discussed under the reaction-mass transport section, Tosenberger et al. make use of DPD to account for the interactions between platelets. The model distinguishes between platelets covered by fibrin and those which are less strongly bound to the clot mass (Filipovic et al., 2008; Tosenberger et al., 2013). Again, the level of detail in terms of platelet interactions could be excessive for the aneurysm model, but the model demonstrates strong coupling between platelet behavior and fluid transport. Storti et al. and Storti and Vosse use Sorenson's model to account for platelet and biochemical activity (Storti and Vosse, 2014; Storti et al., 2014). Rather than altering the fluid properties (porosity or viscosity) to account for the effect of the clot on the flow field, they model the clot as a solid mass and define fluid-structure interaction to couple the behavior of the clot to the flow field. This approach is much more robust and direct, but may come at a higher cost than altering fluid properties. Finally, Stalker et al., Tomaiuolo et al., and Welsh et al. al make use of a systems biology approach to develop a model which emphasizes the changes in molecular transport within the clot region by altering porosity, permeability and effective diffusivity (Stalker et al., 2014; Tomaiuolo et al., 2014; Welsh et al., 2014, 2016). Emphasis is also placed on the core and shell regions of the growing clot. Their experiments demonstrate the accuracy of this approach and implementation in a computational model of cerebral aneurysm thrombosis could be achieved at an acceptable computational cost.

Several models were presented in the preceding discussion. Integrated models would prove easiest to implement in cerebral aneurysms, as they describe the clotting process comprehensively. The greatest setback with these models is that their level of detail could require significant computational resources, especially in patient-derived, three-dimensional geometries. This may not be practical for application in a clinical context. It would therefore prove worthwhile to carry out a sensitivity analysis that could establish crucial modules. The results from such an analysis may show that the simpler models are adequate for the application at hand. A summary of the general physiological models can be seen in Table 3.

**Table 3:** Summary of the physiological clotting models, illustrating the aspects of coagulation that each model incorporates.

**Model**	**Included features**			
	**Biochemistry**	**Haemodynamics**	**Platelet activation**	**Clot mechanobiology**
Hemker/Wagenvoord et al.	×			
Hockin et al.	×			
Filipovic et al.		×	×	×
Chatterjee/Purvis et al.			×	×
Flamm et al.	×	×	×	
Mody and King		×	×	
Wu et al.		×	×	
Basmadjian	×	×		
Pantaleev et al.	×	×		
Anand et al.	×	×		
Bodnar et al.	×	×		×
Sorenson et al.	×	×	×	
Anand et al.	×	×	×	×
Leiderman et al.	×	×	×	×
Xu et al.	×	×	×	×
Tosenberger et al.	×	×	×	×
Storti et al.	×	×	×	×
Welsh et al.	×	×	×	×

### Cerebral aneurysm thrombosis models

In the previous section, models developed to improve current understanding of physiological clotting were presented. These were then compared to each other and assessed for applicability to a cerebral aneurysm thrombosis model. A fair amount of effort has been directed at developing cerebral aneurysm thrombosis models. In this section, these models are presented. With the exception of one model which makes use of an idealized geometry, all use realistic, patient-derived aneurysm geometries. Some modules in these models are borrowed from general physiological models. These are then coupled to flow and applied to aneurysm geometries. The different models used are classified either as thrombosis potential models or as direct thrombosis models.

#### Thrombosis potential models

Several studies make use of hemodynamic and geometric variables as surrogate measures for thrombosis potential. With improved accuracy of computational fluid dynamics methods, a fair amount of detail can be obtained from patient-specific simulations. Variables such as aspect ratio, strain rate and residence time have been studied as potential markers for thrombus development. These values have been linked to thrombosed regions in clinical cases. Several models which follow this approach are presented below.

Sadasivan et al. produced a mathematical model that examines washout from cerebral aneurysms following stent placement (Sadasivan et al., 2002). The model is based on angiographic data from an *in vitro* experiment, and from five patients. The *in vitro* experiment made use of an idealized spherical side wall aneurysm and a pulsatile flow circuit. For both the idealized geometry and patient-derived data, angiography was used to capture flow within the aneurysm and its surroundings, and grayscale intensity curves were generated. These results were then used to develop a mathematical model, and to establish parameters relating to convective and diffusive transport within cerebral aneurysms and their parent vessels. The link between these parameters and stent efficacy was then examined.

Butty et al. presented one of the earliest models that made use of results from a computational fluid dynamics study to correlate residence time with thrombosis potential in realistic, patient-derived cerebral aneurysm geometries (Butty et al., 2002). In their model, two saccular aneurysms on the right internal carotid artery are considered. The Navier-Stokes equations are used to describe flow in the parent artery and in the aneurysm sac, and a finite volume approach is used to discretize and solve the governing equations. In order to speed up convergence, algebraic multigrid acceleration is used. Residence time maps are constructed based on local flow patterns. The significance of the residence time profiles was then discussed in light of thrombosis and pharmacokinetics.

In their studies, Rayz et al. developed the idea presented by Butty et al. and considered flow residence time and wall shear stress as potential markers of thrombus prone regions in cerebral aneurysms (Butty et al., 2002; Rayz et al., 2008, 2010). MRI scans were used to reconstruct patient-derived geometries of cerebral aneurysms. The flow in the aneurysm and parent vessel is described using the Navier-Stokes equations and a finite volume approach is used to discretise and solve the equations. Second-order schemes are employed for spatial and temporal integration. A PISO scheme is used for pressure-velocity coupling and an implicit solver is used for solving the Navier-Stokes equations. A virtual ink technique was developed to monitor flow residence within the region of interest. A passive scalar representing the ink is injected at the inlet of the flow regime. The transport of this “ink” is described and tracked by a convection-diffusion equation. Comparisons were made between computational results and MRI scans.

De Sousa et al. presented a model which investigates the use of geometric and hemodynamic variables as markers for predicting spontaneous and device-induced thrombus growth in cerebral aneurysms (De Sousa et al., 2015). The model makes use of patient-derived cerebral aneurysm geometries segmented from computed tomography angiography for the spontaneous thrombosis cases, and digital subtraction angiography data for the flow diverter cases. To represent the flow diverter within the geometry, a virtual grid was designed and placed inside the parent vessel. The Navier-Stokes equations are used to describe flow in the geometry and a finite volume approach is used to discretize and solve the governing equations. A PISO scheme is used for pressure velocity coupling. Comparisons were made between aneurysm dome to neck ratio, computed strain rate and clot volumes calculated from scans.

#### Direct thrombosis models

Direct thrombosis models combine local hemodynamics with other modules described in the physiological clotting section. Rather than using hemodynamic variables as surrogates for thrombosis potential, the models directly account for biochemical and/or platelet activity, and often account for the impact of the growing clot on the flow field. The models are detailed below.

Bedekar et al. produced one of the earliest computational models of cerebral aneurysm thrombosis (Bedekar et al., 2005). The model accounts for hemodynamics, platelet activity, coagulation proteins and realistic patient-derived anatomies. The modules describing coagulation protein reactions and platelet activity are based on those developed by Sorenson et al. (Sorensen et al., 1999a,b). The main contribution of this model is the coupling of the biochemistry and platelet modules to local hemodynamics in realistic, medical image-derived vascular segments obtained from computed tomography scans. The Navier-Stokes equations are used to account for the hemodynamics in the model and convection-diffusion-reaction equations are used to calculate the changing concentration of the platelet and coagulation species. A finite volume scheme is used to discretize and solve the governing equations. Pressure-velocity coupling is treated with a SIMPLE-C algorithm and convergence is sped up using algebraic multigrid acceleration.

Ouared et al. presented a computational model that developed approaches introduced by thrombosis potential models (Ouared et al., 2008). The key contribution made by this model was that the growing clot affected the flow field, and vice versa. Thrombus growth in the aneurysm sac is governed by a shear regulated mechanism. Below a shear rate of 100 s^−1^, clotting is initiated and growth occurs. A Lattice Boltzmann method for hydrodynamics is used to compute hemodynamics in an idealized aneurysm geometry. The method computes forces experienced by fluid particles in a discrete space-time setup. The particles can move and collide with each other. The local shear rate is computed from the equations and clot growth takes place in computational cells where the shear rate falls below the threshold. The clot region, once formed, is treated as a no-flow-through solid. Using a shear rate threshold makes it possible to account for partial and complete occlusion of the aneurysm sac. The model captures initiation, growth and cessation of a clot.

Ngoepe and Ventikos and Peach et al. presented a model that further developed the contributions made by Bedekar et al. (Bedekar et al., 2005; Peach T. W. et al., 2014; Ngoepe and Ventikos, 2016). The model accounts for hemodynamics and biochemical reactions in realistic, patient-derived, cerebral aneurysm geometries. In addition, the model incorporates flow diverter placement and the effect of the growing clot on the flow field, and vice versa. This model is used to predict clotting outcomes following flow diverter treatment, but highlights the need for model validation before it could be used in clinical studies. The Navier Stokes equations account for fluid flow while convection-diffusion-reaction equations account for the changes in coagulation protein concentrations. The biochemical reactions are based on models developed by Hockin et al. and Wagenvoord et al. and the transport of proteins in the clot region are altered by an effective diffusivity equation (Hockin et al., 2002; Wagenvoord et al., 2006). The continuity and momentum equations are adjusted to incorporate porosity and permeability, allowing for differentiation between the clot and plasma regions. A level set method is used to track the surface of the growing clot. Clot initiation is governed by a shear rate threshold, while clot growth (tracked by the motion of the surface) is linked to thrombin concentration.

Ou et al. also developed the framework introduced by Bedekar et al. and examined fibrin accumulation in cerebral aneurysms following flow diverter placement (Bedekar et al., 2005; Ou et al., 2016). They contest that thrombus development in cerebral aneurysms following flow diverter placement is induced by stasis and developed a model on that basis. Their model includes patient-derived aneurysm geometries, hemodynamics, biochemical equations, the effect of the clot on the flow field, and flow diverter placement. Navier-Stokes equations are used account for the local hemodynamics, and blood is modeled as a non-Newtonian fluid using the Carreau-Yasuda model. The biochemical equations are based on those developed by Anand et al. which were developed for static blood (Anand et al., 2008). These equations are coupled to flow, and convection-diffusion-reaction equations are used to solve species concentrations. Porosity and permeability are adjusted to account for the physical properties of the clot, and to account for the impact of the growing clot on the flow field and vice versa. A fibrin thresholding technique is used to distinguish between clotted regions and normal plasma. If fibrin exceeds a concentration of 350 nM in a specific computational cell, the cell assumes properties of the clot. An experimental rat model was developed to validate the computational model. The right common carotid artery was ligated and thrombus formation was observed. Histological slices were obtained from the experimental model and fibrin concentration was calculated on each slice. This data was then used to validate the computational model.

#### Comparison of different approaches employed in developing computational models of cerebral aneurysm thrombosis

Thrombosis potential studies create a link between hemodynamic variables and thrombus development. An important step in these studies is the comparison between computed hemodynamic values and patient-derived data. In their mathematical model, Sadasivan et al. determined parameters which could delineate the contributions of convection and diffusion in patient-derived arteries with stents (Sadasivan et al., 2002). A direct link was shown between four of the six parameters and stent efficacy. They suggested that the altered hemodynamics (e.g., regions of stasis) following stent placement could indicate high thrombosis potential. At a similar time, Butty et al. developed a computational model that examined flow residence time in patient-derived cerebral aneurysm geometries (Butty et al., 2002). It was found that particles within close proximity of each other had very different residence times. This suggested an association between residence time and thrombosis, even though the link was not clearly understood. Rayz et al. further examined the role of residence time in thrombus development, and also considered wall shear stress as a potential marker (Rayz et al., 2008, 2010). The study found a correlation between regions of increased residence time and low wall shear stress on the computational model, and thrombosis on MRI scans. De Sousa et al. found a strong association between dome to neck aspect ratio of the aneurysm and spontaneous clot volume (De Sousa et al., 2015). Areas of clot growth were also correlated with a strain rate threshold, below which spontaneous clotting was observed. This work corroborated the shear rate threshold hypothesis suggested by Ouared et al. (2008). The ongoing development of thrombosis potential studies is promising and this approach could be effective in clinic as it requires fewer patient-specific input variables and is less computationally expensive when compared to direct thrombosis approaches.

The combination of hemodynamics and biochemistry in realistic, patient-derived cerebral aneurysms geometries was introduced by the work of Bedekar et al. (Bedekar et al., 2005). Until that point, models developed in realistic geometries accounted for hemodynamics only, while those that accounted for the complex biochemistry and platelet environment were implemented in idealized geometries. Ouared et al. take a different approach to that employed by Bedekar (Ouared et al., 2008). The model builds on thrombosis potential models, however, clot growth is tracked over time and the growing clot is modeled as a solid, hence it affects the flow field. A shear rate thresholding technique is used to govern clot initiation, growth, and cessation. Ngoepe and Ventikos, and Ou et al. build on Bedekar et al.'s approach by introducing various levels of sophistication (Ngoepe and Ventikos, 2016; Ou et al., 2016). The main development presented by both groups was that the clot had an impact on the flow field, and vice versa. This was achieved by altering the porosity and permeability in the clot region. In addition, both groups included device placement (flow diverters) in the parent vessel. Ou et al. determine clot growth by a fibrin concentration thresholding technique, while Ngoepe and Ventikos track the surface of the growing clot by considering thrombin concentration in a level set method. In the latter model, clot initiation is governed by a strain rate thresholding technique, building on the approach introduced by Ouared et al. The major advantage of Ou et al.'s model is that it has been validated by studies in an experimental rat model.

Two approaches have been taken when developing computational models of cerebral aneurysm thrombosis. The first approach relies heavily on the accurate computation of hemodynamics within the region of interest. Variables obtained from these calculations are then used to predict thrombus-prone regions. The second approach couples the biochemistry of clotting with hemodynamics and can often account for the clot growth process. For both approaches, using accurate, patient-derived aneurysm geometries has proven crucial as the complexity of the pathology affects local hemodynamics. In addition, it has been shown that including flow diverters in the patient-derived geometries yields different hemodynamic and clotting results, highlighting the need for accurate modeling of devices. The main differences between the two approaches are the ability to capture the temporal evolution of the clot and the ease of validation of the models. The thrombosis potential models are likely to predict the same spatial location for clotting when compared to the direct thrombosis models. If there is interest in how the clot will progress over time to fill that space, the direct thrombosis models are likely to have an advantage. The key consideration when choosing one approach over the other will be the time point at which one wants to examine clotting. If determining outcome immediately after device placement is the main goal, either approach will suffice. If outcome over a longer time period is desired, then direct thrombosis models are more suitable as they account for the different stages of clot development. The main advantage of thrombosis potential models is that direct validation using existing experimental techniques is much easier and results are therefore more reliable. Validation techniques for direct thrombosis models are being developed but more work is required before these techniques become routine (Gester et al., 2016). A summary of the cerebral aneurysm thrombosis models can be seen in Table 4.

**Table 4:** Summary of computational cerebral aneurysm thrombosis models, illustrating the aspects of coagulation that each model incorporates.

**Model**	**Included features**					**Flow diverter**
	**Biochemistry**	**Haemodynamics**	**Platelet activation**	**Clot mechanobiology**	**Medical imaging**	
Sadasivan et al.		×			×	×
Butty et al.		×			×	
Rayz et al.		×			×	
Bedekar et al.	×	×	×		×	
Ouared et al.		×		×		
De Sousa et al.		×			×	×
Ngoepe et al.	×	×		×	×	×
Ou et al.	×	×		×	×	×

## Conclusions

The ideal computational model of thrombosis in cerebral aneurysms would be capable of predicting occlusion outcome for a range of endovascular treatments, in a timeframe with the least impact on overall procedural time. Aneurysm geometries must be accurately represented as these have a significant impact on the variation in outcome between different patients. The model would need to account for the patient's clotting profile and would also need to account for the effect of different treatment regimens. The local hemodynamics also need to be incorporated into the model. This could be achieved through suitable boundary conditions and the assignment of appropriate physical properties to the clot region. Finally, in as far as is possible, the computational framework would need to align with existing facilities at healthcare centers focusing on endovascular treatment of cerebral aneurysms. This would include compatibility with existing imaging modalities and coagulation tests.

Though much has been achieved, there are still several issues to be addressed before the goals described in the previous paragraph can be achieved. The main areas that can be strengthened include computation of hemodynamics, elucidation of the underlying biochemical cascade, clearer definition of occlusion, inclusion of clot maturation and modeling of different endovascular interventions. As pertains to hemodynamics, one of the key areas of focus is developing boundary conditions that are representative yet computationally inexpensive. The biochemical cascade that describes cerebral aneurysm thrombosis has not yet been characterized. Various suggestions and assumptions have been made but few studies have focused on elucidating the exact biological mechanisms at play during the process. Currently, the biochemistry and platelet biology is based on physiological models of clotting. *In vitro* and *in vivo* studies that establish the exact cascade would be invaluable. Clinical observations have shown that usually a clot which occludes the aneurysm sac following the placement of an endovascular device is most desirable. The clinical studies reviewed reported occlusion at different levels and at different time points. It would be beneficial if reporting on occlusion could be standardized. This would make comparisons between different devices easier, both in clinic and for *in silico* models. Clot maturation is a completely unexplored area in cerebral aneurysm thrombosis models. Most models can predict the outcome following intervention but do not account for the longer term period between occlusion and stabilization. While post-intervention clotting outcome is a useful metric, it does not always correlate with longer term stability as the biological mechanisms that govern the two processes are different. It would therefore be beneficial if techniques that model the longer term process were developed. Finally, most models presented have modeled flow diverter placement. To replicate the choices available to a clinician, it would be useful if models accounted for coil embolization and stent-coil combinations. Computational methods which can account for the deployment of different endovascular devices and account for their interaction with the surrounding environment would be of great value. In the long term, clotting patterns emerging from a large aneurysm database may make it possible to develop clearer guidelines for device selection based on aneurysm size and shape.

A review of existing computational models was presented. First, physiological clotting models were described and discussed as these often support cerebral aneurysm thrombosis models. Robust techniques for modeling the clotting process exist. In addition, many models have presented several approaches to coupling the different modules that account for *in vivo* clotting. Some models present mechanisms for linking results obtained from clinical tests to mathematical descriptions of chemical reactions that can be included in an *in silico* model. These are highly desirable features as they account for patient specificity. While integrated models present the highest level of sophistication, it would be worthwhile establishing whether all the features included are necessary for predicting occlusion outcome in a clinical context. Maybe some of the simpler models strike a better balance between detail and computational cost. Most of the physiological models have also been implemented in two-dimensional frameworks only. The extension to three-dimensions would need to be investigated. Existing cerebral aneurysm thrombosis models were also presented and discussed. Two approaches were taken to developing these models, with one focusing on thrombosis potential and the other on direct thrombosis modeling. Almost all the models incorporate patient-derived geometries and complex hemodynamics. Some include biochemistry and device-placement. At this stage, the models are computationally expensive and unsuitable for immediate implementation in clinic. An investigation into the balance between detail and computational cost would also need to be carried out for these models. The section exploring demands placed on clotting models presented various suggestions for simplification, such as simplified boundary conditions and computationally inexpensive methods for representing the physical properties of the growing clot.

*In silico* models could be of use in framing some questions for *in vivo* and *in vitro* studies in a quantitative manner. Once they reach the required level of maturity and robustness, these models can be used for their original purpose of interventional planning. Clinicians could determine likely clotting outcomes for different devices prior to intervention. In addition, device manufacturers could use an *in silico* model during the design process. To make *in silico* models a suitable design and planning tool, the basic parameters which capture the salient features must be determined and computational approaches that minimize computing time must be employed. The review has highlighted the indispensability of biochemistry and fluid dynamics in such models. Once these objectives are achieved, integrative computational tools of the flavor described promise to have substantial impact in minimizing complications and improving clinical outcomes for cerebral aneurysm healthcare.

## Author contributions

MN contributed to concept and design, drafting, critical revision and final acceptance of the work. AF and YV contributed to concept and design, critical revision and final acceptance of the work. JB contributed to critical revision and final acceptance of the work.

### Conflict of interest statement

The authors declare that the research was conducted in the absence of any commercial or financial relationships that could be construed as a potential conflict of interest. The reviewer JB and handling Editor declared their shared affiliation, and the handling Editor states that the process nevertheless met the standards of a fair and objective review.
